# G-Links: a gene-centric link acquisition service

**DOI:** 10.12688/f1000research.5754.2

**Published:** 2015-11-18

**Authors:** Kazuki Oshita, Masaru Tomita, Kazuharu Arakawa

**Affiliations:** 1 Institute for Advanced Biosciences, Keio University, Fujisawa, 252-0882, Japan

**Keywords:** databases, bioinformatics, data integration, molecular biology

## Abstract

With the availability of numerous curated databases, researchers are now able to efficiently use the multitude of biological data by integrating these resources via hyperlinks and cross-references. A large proportion of bioinformatics research tasks, however, may include labor-intensive tasks such as fetching, parsing, and merging datasets and functional annotations from distributed multi-domain databases. This data integration issue is one of the key challenges in bioinformatics. We aim to provide an identifier conversion and data aggregation system as a part of solution to solve this problem with a service named G-Links, 1) by gathering resource URI information from 130 databases and 30 web services in a gene-centric manner so that users can retrieve all available links about a given gene, 2) by providing RESTful API for easy retrieval of links including facet searching based on keywords and/or predicate types, and 3) by producing a variety of outputs as visual HTML page, tab-delimited text, and in Semantic Web formats such as Notation3 and RDF. G-Links as well as other relevant documentation are available at http://link.g-language.org/

## Introduction

The use of large-scale data or multi-domain information is becoming a prerequisite in all fields of molecular biology, in light of the advent of high-throughput measurement technologies exemplified by the new generation DNA sequencers, and further driven by the conceptual progress in integrative systems biology approaches. Typical analysis encompasses multiple genes in a pathway or in a regulatory network, uses orthologous gene sets in related organisms, and merges information from multiple-omics layers such as genome, transcriptome, proteome, and metabolome ( Arakawa & Tomita, 2013). Bioinformatics researchers therefore need to collect and integrate data from a variety of sources, each with diverse syntax, semantics, protocols, identifiers and naming conventions ( Bhagat *et al.*, 2010; Brazas *et al.*, 2012; Katayama *et al.*, 2010). This data integration issue is one of the key challenges in the field of bioinformatics ( Aoki-Kinoshita *et al.*, 2015; Katayama *et al.*, 2014; Stein, 2002; Stein, 2008). While the integration of web services under standardized protocols has seen a sound progress over the last few years ( Katayama *et al.*, 2011), data integration with efficient cross-domain queries still requires the use of large-scale data warehouses such as BioMart ( Smedley *et al.*, 2009) and InterMine ( Smith *et al.*, 2012).

Since the majority of biological databases are well curated with cross-references, related information can be retrieved *ad hoc* from dispersed databases using hyperlinks. In order to facilitate such processes, web services that collect and provide the cross-reference information from different databases ( Diehn *et al.*, 2003; Wu *et al.*, 2013) as well as ID conversion services that assist cross-referencing have been developed ( Cote *et al.*, 2007). MyGene.info, for example, provides rapid programmatic access through a RESTful interface for gene-centric queries to retrieve cross-reference information from numerous databases. Gene-centric aggregation, which integrates databases using genes as the primary key, is a highly efficient approach in molecular biology, since the majority of databases have some sort of connection to genes or proteins, due to the success of the “central dogma” of molecular biology. Ideally, a database should be free from predefined schema or primary keys, and should have controlled syntax and semantics. Semantic Web initiatives are therefore collaboratively aiming to provide such framework through HyperText Transfer Protocol (HTTP) with Resource Description Framework (RDF) and Web Ontology Language (OWL) ( Katayama *et al.*, 2013). For example, the current release of Bio2RDF resource enables integration and federated queries across 35 datasets ( http://download.bio2rdf.org/release/3/release.html). However, the user is required to be familiar with the SPARQL query language, unlike the more intuitive RESTful API approach.

Cross-reference services usually provide database name and identifiers that do not explicitly define the actual location of the data. Moreover, gene-centric data aggregation services usually do not allow querying of gene sets. To this end, here we describe a new RESTful service named G-Links, which gathers Uniform Resource Identifiers (URI) from more than 100 databases in a gene-centric manner, and provide querying interface based on gene sets for hundreds of species. G-Links can be used programmatically as text data, from Semantic Web services, or from graphical HTML pages.

## Implementation

G-Links is implemented with Perl programming language and MySQL 5.0, and has a straight-forward RESTful user interface. The server provides a uniform interface based on URL and HTTP in a client-server model, which is stateless and therefore the server does not store any client context information, and the clients and intermediates can cache responses between server update cycles, duration of which is specified by HTML META tag. G-Links internally resolves cross-references in four steps: ID conversion, retrieval of cross-references, filtering and extraction, and formatting of output. G-Links stores all cross-reference information in a gene-centric manner, and for this purpose, it utilizes UniProt IDs as the primary key. Therefore, G-Links first converts the user input to UniProt ID by ID conversion, based on 80 databases supported by UniProt ID Mapping Service ( Huang *et al.*, 2011). When a nucleotide or amino acid sequence is given as the query, G-Links searches the corresponding UniProt IDs by sequence similarity search using BLAT ( Kent, 2002) against Swiss-Prot database ( Bairoch *et al.*, 2004), and when RefSeq ID for bacterial genomes or taxonomy ID is used as the input, G-Links collects all UniProt IDs of genes within the given species based on UniProt taxonomy ( http://www.uniprot.org/taxonomy/). In the second step, G-Links collects all text annotations and database cross-references about the gene of interest, gathered from over 130 databases. Here the mapping to Gene Ontology slim ( Harris *et al.*, 2004) is pre-computed using map2slim ( http://search.cpan.org/~cmungall/go-perl/scripts/map2slim), and resulting URLs for over 30 RESTful bioinformatics analysis web services supported by the G-language Web Services ( Arakawa *et al.*, 2010) and Keio Bioinformatics Web Service (KBWS) ( Oshita *et al.*, 2011) are generated on-the-fly. KBWS is an European Molecular Biology Open Software Suite (EMBOSS) ( Rice *et al.*, 2000) associated software package for accessing popular bioinformatics web services such as BLAST. All cross-references include the URI of the actual location of data, often expressed as Persistent Uniform Resource Locators (PURLs). Retrieved gene set and annotations are optionally filtered in the third step according to user input, and are formatted in the specified output format in the last step.

## Results and Discussions

**Figure 1.**
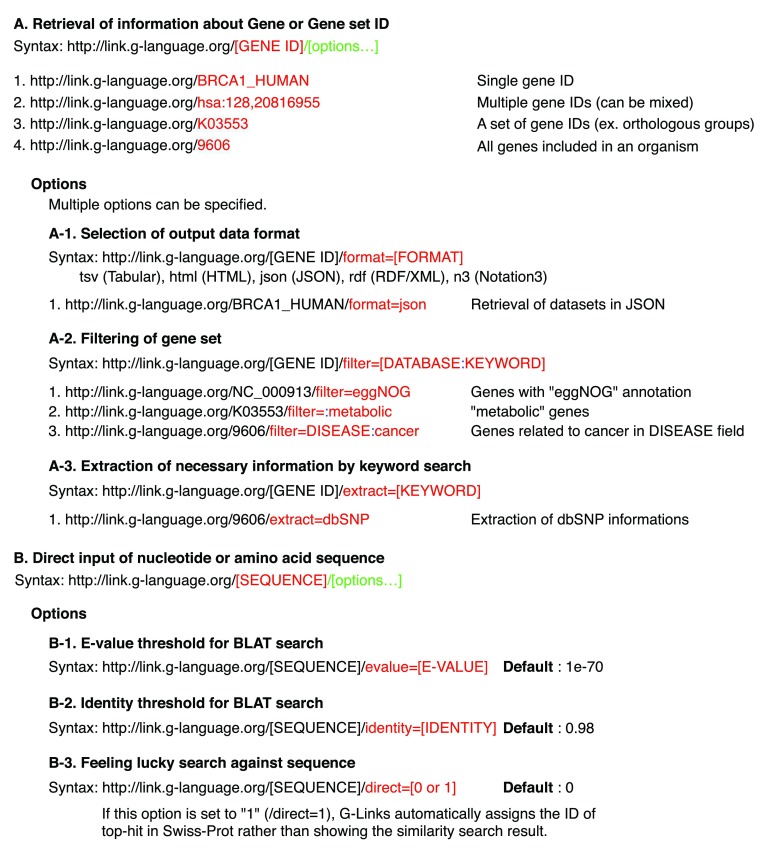
URL Syntax of G-Links. G-Links is implemented as a RESTful service that can be queried by altering the URL. Full documentation and example queries are available at http://www.g-language.org/wiki/glinks.

**Figure 2.**
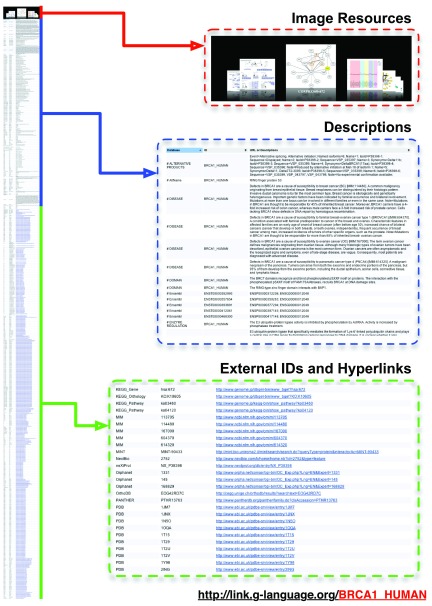
HTML output example of BRCA1_HUMAN (UniProt ID of BRCA1 gene in humans). By default, access to G-Links with web browsers displays the results in interactive HTML, with related image gallery implemented with CoverFlow ( http://imageflow.finnrudolph.de/) on the top, followed by a large table of annotations and cross-references.

G-Links is available at http://link.g-language.org/ as a RESTful web service, which is suited for resource-centric access and highly accessible via HTTP. Users can rapidly retrieve annotations and cross-references related to a given gene ID, taxonomy ID, or raw sequence data by simply accessing a certain URL. An overview of the URL syntax is presented in Figure 1. For example, the URL to retrieve all annotations and cross references related to the human BRCA1 gene (UniProt ID: BRCA1_HUMAN) is simply http://link.g-language.org/BRCA1_HUMAN ( Figure 2). The ID of gene used in this query can be any of the identifiers used in 80 databases supported by G-Links. In this way, multiple URIs can point to the same resource. Programmatic access to this URL can retrieve all 653 annotations and cross-references within 0.2 seconds (tested on dual Xeon X5470 server). G-Links automatically adjusts the output format according to the user context, and outputs the results in human-readable interactive HTML format when accessed from modern HTML browsers, or in Tabular Separated Values (TSV) text format for programmatic access. The HTML format displays a gallery of image resources on the top, such as the pathway maps from KEGG database ( Kanehisa *et al.*, 2012), co-expressed gene network from COXPRESdb ( Obayashi *et al.*, 2013), and protein 3D structure from Protein Data Bank ( Rose *et al.*, 2013), followed by a long table of text annotations and cross-references including database name, ID, and resource URL. Table 1 shows an overview of the categories of databases and web services supported by G-Links output (see http://link.g-language.org/input_list and http://link.g-language.org/output_list for complete listings). In addition to the human-friendly HTML format and computer readable TSV as well as JavaScript Object Notation (JSON) output, G-Links supports RDF/XML and Notation3 ( http://www.w3.org/TeamSubmission/n3/) formats, so that the query results can be readily integrated with Semantic Web technologies. For RDF and Notation3 predicate information is given by EMBRACE Data and Methods (EDAM) ontology.

**Table 1.** Overview of supported databases and web services in G-Links. Detailed list of Input/Output databases are available at http://link.g-language.org/input_list and http://link.g-language.org/output_list.

Databases (132)				
Genome(11)	Phosphorylation(3)			
Gene(6)	Ortholog(7)	Cluster(1)	Expression(4)	
		SNP(2)	Phylogenesis(2)	
Protein(4)	Structure(5)	Classification(1)	Cluster(4)	
	Family/Domain/Motif(9)	PPI(4)	Enzyme(3)	
Molecular Interaction(2)	Pathway/Reaction(5)	DISEASE/Pathogen/Drug(6)		
Others(15)	Paper(3)	Organisms specific(31)		
Web Services (33)				
	Alignment Local(1)	Data Retrieval Chemistry Data(1)		
	Nucleic Composition(5)	Nucleic CpG Islands(1)	Nucleic Translation(1)	Nucleic Repeats(3)
	Protein Properties(5)	Protein 2D Structure(3)	Protein Composition(3)	Protein Motif(3)
	Protein Localization(4)	Protein Domains(2)	Protein Functional Site(1)	

Likewise other bioinformatics tools such as BioGrid ( Chatr-Aryamontri *et al.*, 2015) and Cytoscape ( Demchak *et al.*, 2014), G-Links can retrieve information related to gene sets or all genes of organisms, and to filter out non-related genes by keyword search ( *filter* option) or to extract necessary fields ( *extract* option). Using the filtering option, users can retrieve only the subset of genes related to the given keyword. For example, retrieval of all human (taxonomy ID: 9606) genes having GO slim function including the word “transport” is as simple as http://link.g-language.org/9606/filter=GOslim_function:transport/format=tsv/. Similarly, extraction of only the “DISEASE” annotation of BRCA1 gene is simply http://link.g-language.org/BRCA1_HUMAN/extract=DISEASE. Multiple filtering and extraction conditions can be specified using “|” (vertical bar) as the separator, in order to formulate complex queries. For example, retrieval of SNP information from dbSNP and SNPedia for human genes with known polymorphisms related to breast and ovarian cancer in tabular format is queried as http://link.g-language.org/9606/format=tsv/filter=DISEASE:cancer/filter=:breast|:ovarian|:snps|:polymorphisms/extract=dbSNP|SNPedia.

The gene-centric approach is effective for data aggregation from a variety of databases, especially for prokaryotes, where the genes, transcripts, and proteins are mostly synonymous. On the other hand, this approach can be a limitation for many questions in eukaryote systems biology that require a transcript-centric approach due to the large complexity and diversity of transcriptome regulated by alternative splicing ( Nilsen & Graveley, 2010). Currently G-Links lists information of all transcript isoforms, their structures and other annotations, and therefore the gene-centric information can be queried from the identifiers related to the isoforms, but not necessarily the other way around.

## Conclusions

By serving as a data hub of linked open biological data, G-Links can be a starting point in retrieval of gene-centric information. Users can quickly obtain related links and annotations of a gene of interest either graphically via HTML or programmatically via REST interface, such as the orthologs, Gene Ontology terms, protein structure, pathways, SNPs, and publications.

## Software availability

### Software access

G-Links is a RESTful service with base URL http://link.g-language.org/. Detailed documentation is available at http://www.g-language.org/wiki/glinks including service description, syntax, list of all available options, example queries (URLs) and sample scripts for programmatic access in Perl, Ruby, Python, and Java. Examples of programmatic access from the UNIX commandline for Gene Ontology classification of all genes in *E. coli*, as well as for specific set of genes of interest for possible Gene Ontology enrichment analysis, or KEGG BRITE enrichment analysis are also provided. Comprehensive lists of supported input/output databases and web services are available at http://link.g-language.org/input_list and http://link.g-language.org/output_list. Internal database of G-Links is regularly updated every six months, and only the latest version of each resource is accessible, and the source code is freely available from GitHub repository ( http://github.com/cory-ko/G-Links).

### Latest source code

http://github.com/cory-ko/G-Links

### Source code as at the time of publication

https://github.com/F1000Research/G-Links/releases/tag/v1.0

### Archived source code as at the time of publication

http://dx.doi.org/10.5072/zenodo.12701 ( Oshita & Arakawa, 2014).

### License

MIT License
