# Supplementary material for: G-Links: a gene-centric link acquisition service
Source: F1000Res. 2015 Nov 18;3:285. Originally published 2014 Nov 19. [Version 2] doi: 10.12688/f1000research.5754.2 (PMC4670005; doi:10.12688/f1000research.5754.2)
Supplement: Peer review history

# Peer review: Referee response for version 2

**DOI:** 10.5256/f1000research.6534.r11277

**Published:** 2015-11-20

**Authors:** Mark Ragan

**Affiliations:** 1 Institute for Molecular Bioscience, The University of Queensland, St Lucia, QLD, 4072, Australia

The Authors have responded satisfactorily to our queries and concerns.

I have read this submission. I believe that I have an appropriate level of expertise to confirm that it is of an acceptable scientific standard.

# Peer review: Referee response for version 1

**DOI:** 10.5256/f1000research.6153.r7340

**Published:** 2015-01-22

**Authors:** Kenji Satou

**Affiliations:** 1 Institute of Science and Engineering, Kanazawa University, Kanazama, Japan

As described in this paper, G-Links system provides a sophisticated way of accessing gene-related information scattered in various databases. The revisions recommended by the first reviewer are still helpful. I think this paper is worth indexing after following the recommended revisions as much as possible. 

Minor comment: Isn't the number 22681029?

I have read this submission. I believe that I have an appropriate level of expertise to confirm that it is of an acceptable scientific standard.

# Response

**Published:** 2015-11-04

**Authors:** Kazuharu Arakawa

**Affiliations:** Keio University, Japan

**Minor comment: Isn't the number 22681029?**

The KAKENHI Grant Number is revised accordingly.

# Peer review: Referee response for version 1

**DOI:** 10.5256/f1000research.6153.r6750

**Published:** 2014-11-27

**Authors:** Mark Ragan, Sriganesh Srihari, Alison Anderson

**Affiliations:** 1 Institute for Molecular Bioscience, The University of Queensland, St Lucia, QLD, 4072, Australia

This paper introduces a simple approach to data integration that can assist bioinformatics researchers. The RESTful API is easy-to-use and allows gene-centric linking of information from a very large numbers of data sources.

We recommend the following revisions:

*Scope*

The authors rightly present data integration as a key challenge in bioinformatics (Abstract), and present their work as aiming to solve this problem.  However, data integration is a much deeper problem than just data aggregation.

*Literature review*

The authors might mention earlier link-based aggregators, *e.g.* BioMOBY.

*Gene centricity*

A gene-centric approach is appropriate for some organisms (notably bacteria and archaea) and problems, but many questions in eukaryote systems biology require a transcript-centric approach.  The authors might mention this (significant) limitation.

*Aliases and catching errors*

AOF2 is an alias for KDM1A. A search for KDM1A works fine. A search for AOF2 using the search box on the website or using http://link.g-language.org/AOF2  returns a blank page with no error message. A search using http://link.g-language.org/AOF2_HUMAN gives an inappropriate error message (contact root@localhost).

The article should describe whether and how the system tries to deal with gene aliases, and appropriate feedback should be given to the user when no results are found.

*Website*

G-Links appears to be an extension of the G-language Project. Having the G-Links homepage within the G-language Project wiki is confusing, as menu-items on the left are relevant to the latter.  We also suggest that the ‘Quick Star’ should be near the top of the page to facilitate ease of use.

*Comments on current Semantic Web technologies*

The last sentence of the second paragraph of the Introduction states that with current Semantic Web technologies, cross-domain queries require extensive reasoning or manual curation of ontologies.  This is not an accurate description of the current state of Semantic Web technologies.  The authors might alternatively make reference to the latest version (July 2014) of the Bio2RDF resource, which enables integration and federated queries across 35 datasets, and suggest that a limitation (in comparison to their RESTful API approach) is that the user is required to be familiar with the SPARQL search language.

*Example use cases*

The use cases provided in the manuscript are quite simple and don’t really demonstrate why the G-Links approach can be more powerful than using (for instance) GeneCards to get an overview of a gene such as BRCA1, or just going to the KEGG website and running a search on ‘cancer’.  A more comprehensive use case that shows, for example, how easily results from a gene list search in (for instance) TSV format can be programmatically searched to identify common elements for the genes of interest, might better demonstrate the utility of G-Links.

*Data integration as a current challenge*

The references given to support the claim that data integration is currently a major challenge for the bioinformatics field are 12 and 6 years old. Several features claimed as advantages for G-Links (linking to multiple databases, retrieving information on genes from multiple organisms, filtering by keywords, extracting by fields) are reasonably common, *e.g.* in BioGrid or Cytoscape.

*Licensing*

If any linked resources have license rights that need to be adhered to, this information should be brought to the user’s attention, perhaps using a note on the G-Links website.

*Constraints*

For best practice, the system should meet the constraints for RESTful – stateless, cacheable and so on; these might be described briefly in the paper.

The authors might mention that multiple URIs can point to the same resource.

A resource can exist in different versions; does the system capture and display the version from which information has been captured?

Finally, we recommend that the authors present a Semantic Web-specific example for their statement that ‘G-links can be used programmatically as text data, from Semantic Web services..’.

We have read this submission. We believe that we have an appropriate level of expertise to confirm that it is of an acceptable scientific standard, however we have significant reservations, as outlined above.

# Response

**Published:** 2015-11-04

**Authors:** Kazuharu Arakawa

**Affiliations:** Keio University, Japan

We would like to thank the reviewer for thorough review, and apologize for the extreme delay in our revision. Following are point-by-point comments for our revision.

***Scope***

**The authors rightly present data integration as a key challenge in bioinformatics (Abstract), and present their work as aiming to solve this problem.  However, data integration is a much deeper problem than just data aggregation.**

We have toned down this claim as follows: “We aim to provide an identifier conversion and data aggregation system as a part of solution to solve this problem”.

***Literature review***

**The authors might mention earlier link-based aggregators, *e.g.* BioMOBY.**

BioMOBY is a registry of bioinformatics web services, and is not a link-based aggregators. It allow the suggestion and discovery of web services based on the data type (ex. to discover BLAST services from a FASTA file – discovery of a service for a given input, or NCBI data retrieval services that produces a FASTA file – discovery of a service for a given output.). As an example of earlier link-based aggregators, we have mentioned MyGene.info service.

***Gene centricity***

**A gene-centric approach is appropriate for some organisms (notably bacteria and archaea) and problems, but many questions in eukaryote systems biology require a transcript-centric approach.  The authors might mention this (significant) limitation.**

We thank the Reviewer for this important comment. We have added the following paragraph in the manuscript to clarify this limitation.

“The gene-centric approach is effective for data aggregation from a variety of databases, especially for prokaryotes, where the genes, transcripts, and proteins are mostly synonymous. On the other hand, this approach can be a limitation for many questions in eukaryote systems biology that require a transcript-centric approach due to the large complexity and diversity of transcriptome regulated by alternative splicing {Nilsen, 2010 #178}. Currently G-Links lists information of all transcript isoforms, their structures and other annotations, and therefore the gene-centric information can be queried from the identifiers related to the isoforms, but not necessarily the other way around.“

***Aliases and catching errors***

**AOF2 is an alias for KDM1A. A search for KDM1A works fine. A search for AOF2 using the search box on the website or using** **http://link.g-language.org/AOF2** **returns a blank page with no error message. A search using** **http://link.g-language.org/AOF2_HUMAN** **gives an inappropriate error message (contact root@localhost).**

The system is modified to provide 404 and 500 HTML errors with corresponding error messages when ID cannot be resolved. Redirection for aliases are likewise revised, and now http://link.g-language.org/AOF2_HUMAN shows a list of redirections.

**The article should describe whether and how the system tries to deal with gene aliases, and appropriate feedback should be given to the user when no results are found.**

Error handling is updated as described above. We do not specifically implement support for gene symbols, but UniProt aliases are now correctly handled. For gene symbols, ChiTaRS database contains the information for model eukaryotes, so all information about KDM1A can be retrieved from http://link.g-language.org/KDM1A, although it takes a little while to load all information.

***Website***

**G-Links appears to be an extension of the G-language Project. Having the G-Links homepage within the G-language Project wiki is confusing, as menu-items on the left are relevant to the latter.  We also suggest that the ‘Quick Star’ should be near the top of the page to facilitate ease of use.**

The website ( http://link.g-language.org/) is now not redirected and is given a dedicated page, and the ‘Quick Start’ menu is moved to the top as suggested.

***Comments on current Semantic Web technologies***

**The last sentence of the second paragraph of the Introduction states that with current Semantic Web technologies, cross-domain queries require extensive reasoning or manual curation of ontologies.  This is not an accurate description of the current state of Semantic Web technologies.  The authors might alternatively make reference to the latest version (July 2014) of the Bio2RDF resource, which enables integration and federated queries across 35 datasets, and suggest that a limitation (in comparison to their RESTful API approach) is that the user is required to be familiar with the SPARQL search language.**

Revised accordingly.

***Example use cases***

**The use cases provided in the manuscript are quite simple and don’t really demonstrate why the G-Links approach can be more powerful than using (for instance) GeneCards to get an overview of a gene such as BRCA1, or just going to the KEGG website and running a search on ‘cancer’.  A more comprehensive use case that shows, for example, how easily results from a gene list search in (for instance) TSV format can be programmatically searched to identify common elements for the genes of interest, might better demonstrate the utility of G-Links.**

We now provide illustrative examples of programmatic access from the UNIX commandline for Gene Ontology classification of all genes in *E.coli*, as well as for specific set of genes of interest for possible Gene Ontology enrichment analysis, or KEGG BRITE enrichment analysis in the website, and it is also mentioned in the text.

***Data integration as a current challenge***

**The references given to support the claim that data integration is currently a major challenge for the bioinformatics field are 12 and 6 years old.**

Two more latest reviews are added.

**Several features claimed as advantages for G-Links (linking to multiple databases, retrieving information on genes from multiple organisms, filtering by keywords, extracting by fields) are reasonably common, *e.g.* in BioGrid or Cytoscape.**

We have removed the mention that these are advantages, and mentioned that these features are common as in BioGrid and Cytoscape.

***Licensing***

**If any linked resources have license rights that need to be adhered to, this information should be brought to the user’s attention, perhaps using a note on the G-Links website.**

The information is added to the website.

***Constraints***

**For best practice, the system should meet the constraints for RESTful – stateless, cacheable and so on; these might be described briefly in the paper.**

Brief description is added to the manuscript: “The server provides a uniform interface based on URL and HTTP in a client-server model, which is stateless and therefore the server does not store any client context information, and the clients and intermediates can cache responses between server update cycles, duration of which is specified by HTML META tag.”

**The authors might mention that multiple URIs can point to the same resource.**

Revised accordingly.

**A resource can exist in different versions; does the system capture and display the version from which information has been captured?**

G-Links only provides the latest resource, and the manuscript is revised to include this information.

**Finally, we recommend that the authors present a Semantic Web-specific example for their statement that ‘G-links can be used programmatically as text data, from Semantic Web services..’.**

Since the use of RDF data requires storage of data obtained from G-Links in a triple store with other semantic web resources, we have removed the claim “from Semantic Web services”.
